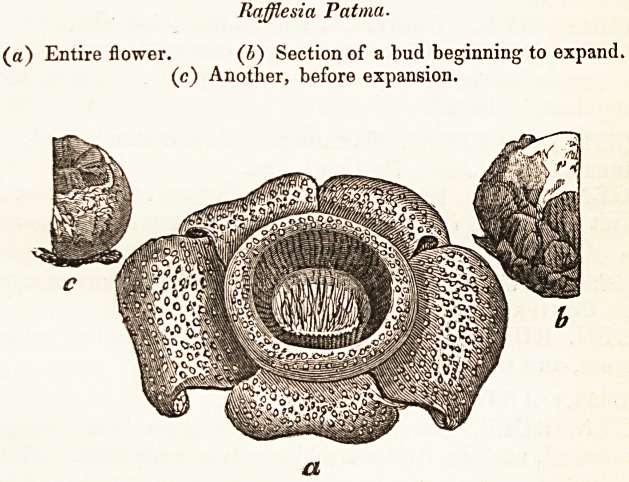# Outlines of Botany; Including a General History of the Vegetable Kingdom, in Which Plants Are Arranged According to the System of Natural Affinities

**Published:** 1835-04-01

**Authors:** 


*Outlines of Botany; including a General History of the Vegetable Kingdom, in which Plants are arranged according to the System of Natural Affinities.* By GILBERT T. BURNETT, F.L.S., Professor of Botany in King's College, London.—London, 1835. Two vols. 8vo. pp. 1190.

IN our first Number we performed the agreeable duty of introducing the early parts of this masterly work to our readers: we have now the still greater satisfaction of announcing that it is completed, and of recommending those alike who may be called the students of botany, and those who merely embellish their leisure hours with the amenities of the most delightful of the sciences, to procure the unrivalled Outlines of Professor Burnett.

To him who aspires to be a botanist, it is quite unnecessary to recommend these well-stocked volumes, for he possesses them already; but we would suggest to the practitioner of physic, who scorns to be a mere dealer in prescriptions, and who knows the grace as well as the light which the kindred sciences throw over medicine, that the work before us deserves a place in the most select library.

We approve highly of our author's practice of intermingling historical and pharmaceutical facts with the botanical definitions; and we should hold ourselves guilty of an illegal clipping of praise, if we were to use the ordinary phrase on these occasions, and merely laud the Professor for "relieving the dry details of botany" by these comments: they seem to us an essential part of the book.

Those publishers of schemes, and tables, and conspectuses, whose works bear the same relation to books that a skeleton does to a body, and whose marrowless productions, dry as the leaves in autumn, seem, like them, destined only to fall to the ground, utterly mistake the final object of the science they would teach. It is true that the study of botanical definition, with its rapid alternations of analysis and synthesis, has a manifest tendency to improve the powers of reasoning; but this is an advantage picked up by the way. The ultimate scope of the science is to teach the properties of plants; and this can hardly be done without at the same time qualifying the student to understand the facts of the historian and the allusions of the poet.

The very names of plants, when affixed by some great master of the art of nomenclature, are not arbitrary symbols. When Linnæus is the nomenclator, we no longer ask, with the poet, "What's in a name?" for the very name tells. How elegant is the allusion in *Salix Babylonica!* how quiet the sarcasm in *Buffonia tenuifolia!* We are glad that Professor Burnett's work affords us many opportunities of illustrating our theory by examples.

Who has not heard of the Upas of Java, that vegetable demon, to approach which was death? It is curious that the fables about this tree did not spring up in the brain of an Italian romancer, but were the calm invention of a plain, sober, poly-bracciferous Dutch surgeon, one Foersch, who wrote some fifty years ago. In 1774, he was stationed at Batavia, in the service of the Dutch East India Company, and, having heard of the violent effects of the Upas poison, his curiosity was raised to so high a pitch, that he determined to investigate the subject personally. Observe how circumstantial the knave is.

"I procured a pass to travel through the island from the governor-general, and a recommendation from an old Malayan priest to another priest who lived on the nearest habitable spot to the tree, which is about fifteen or sixteen miles distant, and who is appointed by the emperor to reside there, in order to prepare for eternity the souls of those who, for different crimes, are sentenced to approach the tree, and to procure the poison.

"The Bohun Upas is situated in the island of Java, about twenty-seven leagues from Batavia, fourteen from Sonra Charle, the seat of the emperor, and between eighteen and twenty from Tinkjor, the present residence of the sultan of Java. It is surrounded on all sides by a circle of high mountains and hills, and the country round it, to the distance of ten or twelve miles from the tree, is entirely barren. Not a tree nor a shrub, nor even the least plant of grass, is to be seen. I have made the tour all round the dangerous spot, at about thirteen miles distant from the centre, and I found the aspect of the country on all sides equally dreary. The easiest ascent of the hill is from the part where the old ecclesiastic dwells. From his house the criminals are sent for the poison, into which the points of all warlike instruments are dipped. It is of high value, and produces a considerable revenue to the emperor.

"The poison which is procured from this tree is a gum that issues out between the bark and the tree itself, like the camphor. Malefactors, who for their crimes are sentenced to death, are the only persons who fetch the poison; and that is the only chance they have of saving their lives. After sentence is passed upon them by the judge, they are asked in court whether they will die by the hands of the executioner, or go to the Upas-tree for a box of poison. They commonly prefer the latter alternative, as there is not only some chance of preserving their lives, but also a certainty, in case of their return, that provision will be made for them in future by the emperor. They are provided with a silver or tortoiseshell box, into which they are to put the poisonous gum, and are properly instructed how to proceed while they are upon the dangerous expedition. Among other particulars, they are always told to attend to the direction of the wind, as they are to go to the tree before the wind, so that the pestilential smell may be blown from them; they are told likewise to travel with the utmost dispatch, as that is the only method of effecting a safe return. They are afterwards sent to the house of the priest, to which place they are commonly attended by their friends and relations; here they generally remain for some days, in expectation of a favorable breeze, during which the ecclesiastic prepares them for their future fate, by prayers and admonitions. When the hour of their departure arrives, the priest puts on them a long leathern cap, with two glasses before their eyes, which generally comes down to the breast, and also provides them with a pair of leathern gloves: they are then conducted by the priest, and their friends and relations, about two miles on their journey. Here the priest repeats his instructions, and tells them where they are to find the tree; he shews them a hill which they are to ascend, and tells them that on the other side they will find a rivulet, which they are to follow, and which will conduct them directly to the Upas; they now take leave of each other, and, amidst prayers for their success, the delinquents hasten away.

"The worthy old ecclesiastic has assured me that during his residence there, for upwards of thirty years, he had dismissed about seven hundred criminals in search of poison, and that scarcely two out of twenty have returned. He shewed me a catalogue of the unhappy sufferers, with the dates of their departure. I was present at some of the melancholy ceremonies, and desired different delinquents to bring with them some pieces of the wood, or a small branch, or some leaves of the wonderful tree. I have also given them silk cords, desiring them to measure its thickness. I never could procure more than one or two dry leaves, that were picked up by one of them on his return; and all that I could learn of him was, that the tree stood on the bank of the rivulet, that it was of a middling size, that five or six young trees of the same kind stood close by it, but that neither shrub nor plant could be seen near it; and that the ground was of a brownish sand, full of stones, almost impracticable for travelling, and covered with dead bodies." (P. 552.)

And then the ingenious Hollander goes on to say, that no living animal of any kind has ever been discovered within the space of fifteen or eighteen miles round the tree; and that, in 1776, he saw thirteen of the sultan's wives put to death by the poison.

"Thus far the historical romance. The facts ascertained by different travellers, and confirmed on many hands, are the following The *Antiar* or *Bohun-Upas*, is a native of Java and the neighbouring isles, growing to a large size, and being found not in barren districts, but in the most fertile places. So far from destroying other vegetables, climbing plants twist round its stem as they do round other trees; neither are its exhalations so noxious as to destroy birds flying over, or animals that approach it; yet, although neither MM. Deschamps and Leschenault experienced any inconvenience, other persons are said to suffer from headach, and to have uncomfortable sensations when in its vicinity, similar to those which are produced by the exhalations of the *Manchineel tree*, the *Rhus radicans*, and other plants, especially some of the *Euphorbiaceæ*. Leschenault even smeared some of the venomous juice over his hands with impunity, but he washed them immediately afterwards. The sap which exudes from wounds made in the tree is a bitter gum-resin. It is of a light hue when drawn from the young branches, and dark yellow if taken from the old stem, but both kinds become nearly black on drying. The Javanese make a mystery of its preparation, and pretend that the fresh sap is inert, and that it gains its power by certain additions they make to it, and the process it undergoes. But Hoosfield has shewn that these pretensions are false. In Java the poison is kept in a semi-fluid state, resembling treacle, while in Borneo it is rendered solid. It is usually preserved in the hollow joints of the bamboo, and, if excluded from the air, retains its extraordinary powers for an unlimited time." (P. 554.)

It would seem that, in Javanese, *Upas* signifies poison, and the island contains a *Upas* valley, as well as a Upas tree. The valley is a huge reservoir of carbonic acid gas, alike destitute of vegetation, and destructive of animal life.

"The origin of Foërsch's centaurian tale must now be evident: that UPAS meant poison, and was an adjective term applied to deleterious things of various kinds, whether trees or places, he knew not; he had heard of the *Upas*, had probably witnessed its effects as a poison, and not improbably had seen the real Bohun Upas tree, which perhaps may sometimes grow in a barren district, such as he has described. He had heard of the Valley of Death, the Upas valley, and he might even have ridden round some sterile spot for thirty miles, fearing to tread upon its precincts, lest he should approach too closely to the chimera he had formed, by combining the accounts of the *Upas valley* and the *Upas tree*." (P. 557.)

To show the varied information with which the Outlines abound, we will now make half-a-dozen short extracts from different parts of the book.

"(3415.) *Cicuta virosa*, the *Cowbane*, is a very poisonous plant to men, and some animals, such as kine; although others, such as horses, sheep, and goats feed on it with impunity. In the moist pastures of Sweden it used to occasion a yearly plague amongst horned cattle, until the cause was pointed out and a preventive suggested by Linneus. When full grown, the odour is so strong that the cows avoid it ; but when young, the smell is so faint that they eat it indiscriminately with the other herbage amongst which it abounds. Linneus therefore recommended the graziers to keep their cattle in the upland pastures until the cowbane was well grown, and then they might be driven to the lowlands, as their instinct wound prevent them touching the plant; his advice was taken, and their annual losses, which were immense, from that period ceased." (P. 775.)

"(3912.) *Sinapis nigra*, and *alba*, are the black and white mustards of commerce. Other species are acrid and pungent, such as the *S. arvensis*, the *corn mustard* or *charlock*, but less so, or of a less agreeable favour, than the two which are in common cultivation. The mustard seeds consist of mucilaginous and farinaceous matter, combined with a bland fixed oil, and a volatile or essential one of great pungency. The acridity of this latter is increased by keeping the seeds for a moderate time after collection, or at once developed by the addition of vinegar. The fixed oil expressed from the seeds of the white mustard is bland and tasteless, while the marc or cake left, after the expression, being deprived of so much mild insipid matter, is more acrid than the seeds in their original condition. The oil is excellent for all ordinary domestic purposes. Nitrogen exists in the seeds as well as other parts of these plants, whence the presence of ammonia and ammoniacal salts, and their peculiar animal odour, may be easily accounted for. White mustard seeds have at different periods been popular as stimulating cathartics, and in leucophlegmatic habits the taking one or two tablespoonsful of the unbruised seeds would seem to have been beneficial; for, in their passage through the intestines, they give out but a small proportion of their pungent principles, and these are obtunded by the mucilage with which they are combined: ulceration of the intestines and death have, however, been known to occur from some of these acrid seeds lodging in the vermiform appendix of the cæcum. A case of fatal enteritis, thus produced, has been recorded by my friend, Professor Wheeler, in his Chelsea Catalogue. The seeds of mustard are not only remarkable for the rapidity of their development, so that it has been said a salad might be grown while a joint of meat was being roasted, but also for their tenacity of life, for where a crop of mustard has been once seeded, self-sown stragglers will come up for a century afterwards." (P. 865.)

"(4047.) The *Coriariæ* are astringent plants, and their leaves, especially those of *C. myrtifolia*, have been employed by dyers to strike a black colour with the salts of iron. Their succulent fruits are, if eaten in any quantity, poisonous. Sauvages witnessed death ensue in half an hour after some were eaten. And Pujada mentions an instance of fifteen soldiers who were poisoned by them in Spain; twelve of these men recovered, but three died. Many other cases are on record; and it appears that a kind of drunkenness is at first produced, which lasts for about half an hour; the face then becomes pale and livid, the speech is lost; there is foaming at the mouth, spasms of the muscles of the jaws, and horrible convulsions of the whole frame, death ensuing in about seventeen hours. The leaves and young twigs, possess the same deleterious properties as the fruit, and when animals browse on them they are seized with intoxication attended by vertigo, and, if much has been eaten, by death. Accidents have happened in France from the leaves of this plant having been fraudulently substituted for senna, and administered to the sick instead of that drug. Guibourt and Dublance detected this iniquitous fraud, their attention having been directed to the circumstance of untoward symptoms following the exhibition of what was believed to be senna. One of the cases on record is that of a man who was seized at Hazebrouk with tetanus, after taking a small quantity instead of senna, and who died in four hours; the remains of the dose were given to a dog, which was killed by it in ten minutes. M. Fee has furthermore stated, that when he visited the drug-warehouses at Lisle, Turcoing, Menin, and their vicinities, in 1828, he found the senna almost universally adulterated with the leaves of the *Coriaria myrtifolia*, called in France *Redon* or *Redoul*. The detection of such frauds is however easy, as the leaves of the *Redoul* differ in their venation from those of the sennas, the basal costules being very long, divergent, and forming an extended intro-marginal line, instead of being equal with the other ribs. The leaves are also pointed, which they are not in the best senna (*Cassia obovata*), but this will not distinguish them from *C. acutifolia*, although to a practised eye the difference in form is obvious, and the venation alone is a sufficient guide." (P. 887.)

"None of the *Labiatæ* are poisonous, nor are any even suspected of being injurious; the betony is the most acrid of the whole. Scarcely any are used as ordinary food, although many form grateful condiments; the stachys and the basil being perhaps the only ones that are esculent as pot-herbs. They are all more or less fragrant, most are sweet-scented, but some are fœtid. Their odours are in general owing to the essential oils which are secreted in abundance, and found in numerous receptacles on their leaves and stalk. Fee observes, that odoriferous plants exhibit three remarkable variations; in some the aromatic principle is free, and then it is dissipated by drying; this occurs chiefly in flowers, such as in the tuberose and jasmine; it is not communicable either to water or spirit, and seems to be artificially retained only by the aid of fixed oils; and occasionally, as in the lilly and narcissus, it cannot be retained at all. In some the aromatic principle is in union with, or is peculiar to the essentially oil, with which the utricles or cryptæ are replete; and in this form it is miscible with water and alcohol, but scarcely with fixed oils. In others again, it is in combination with a resin or gum-resin, and then it may be collected in concrete masses by wounding the plants, or if by distillation, it deposits camphor after standing for some time. The fragrance of the *Labiatæ* is dependent on an essential oil or odoriferous principle of the latter kind, and their oil is remarkable for the quantity of camphor it contains. Besides the essential oils which render them stimulating, the *Menthaceæ* likewise contain a bitter principle, which occasionally is so predominant as to render them useful tonics, and even serviceable febrifuges." (P. 970.)

"(4609.) *Caoutchouc* has been found in the milky saps of several of these plants. It exists in a small but notable quantity in the juices of *Apocynum Androsæmifolium*, *A. Cannabinum*, *Willughbæa scandens*, *Vahea gummifera*, *Urceola elastica*, and others, but more especially in the two last-named, from which it is procured for commercial purposes in great abundance, the chief of the *caoutchouc* brought from the East Indies being extracted from these plants, which are natives of Sumatra and Madagascar. It might probably be obtained from several species of *Tabernæmontana*, to which genus both *Vahea gummifera* and *Urceola elastica* are referred by Sprengel; as well as from various other more or less common plants; for one of the not least remarkable features in the history of this extraordinary substance (once regarded only as a curiosity, and brought to this country in very small quantities as a rarity,) is, that as uses for it have been devised new sources of it have been discovered; and the more its importance and general applicability in the arts have been established, the more common and abundant it has become. Of its numerous applications this is not the place to treat in detail, but it may not be irrelevant to observe, so great is its present consumption, that several thousand tons of it have been imported during the few early months of the current year, while, five or six years ago, it scarcely formed a noticeable entry in our books of customs; and, half a century back, its existence was scarcely known. The first public mention of *caoutchouc*, or, as it was then called, Indian-rubber, which name it still retains, although it is now but seldom used by artists, is in a note, added by Dr. Priestley to the Preface of his "Treatise on the Theory and Practice of Perspective," dedicated to Sir Joshua Reynolds, and published in 1770. He says, "Since this work was printed off I have seen a substance excellently adapted to the purpose of wiping from paper the marks of a black-lead pencil. It must therefore be of singular use to those who practise drawing. It is sold by Mr. Nairne, mathematical instrument maker, opposite the Royal Exchange. He sells a cubical piece of half an inch for three shillings, and he says it will last several years." Now it is imported by tons, and sells at from 2*d.* to 6*d.* per lb.

"(4610.) *Caoutchouc* is a most extraordinary substance, not only in its uses, but also in its chemical composition. It consists solely of carbon and hydrogen, the former in great excess; and, by subjecting it merely to the action of heat, it assumes various different mechanical conditions, and changes its state from a solid to a permanent, and even ethereal liquid form, without varying the proportions of its elements. When once solidified from the sap, in which it is suspended or combined, it is scarcely soluble in any ordinary menstrua, except the essential oils and coal naphtha; but the fluid *caoutchucine*, procured by the distillation of solid *caoutchouc*, is one of its most perfect and effectual solvents; thus affording the remarkable paradox of a substance being soluble in itself. The saps in which it is naturally found in a fluid state are no less extraordinary than the *caoutchouc* they contain, for this solid matter exists in them in a fluid state in the proportion of nearly, if not quite one half of their weight; and yet this sap moves through the delicate vessels of the plants without interruption, thus strangely burdened, but not encumbered. No blood of animals, or any other vital fluid, is known to contain such a vast proportion of solid matter." (P. 1014.)

"(4877.) Two further generalizations cannot however with propriety be omitted. In the first place, it is remarkable that those genera or species which are common to the torrid and temperate, to the temperate and frigid, or to all the several zones, are found of a larger size, and often assume an arborescent port, in the warmer regions; while in the extratropical and circumpolar latitudes they are reduced in size, often degenerating into shrubs or herbs. Thus the Araliaceæ are arborescent plants, the common Umbelliferæ herbaceous ones; the equatorial Asteraceæ trees or shrubs, those of the temperate zones undershrubs or herbs, and those of the circumpolar regions, altogether lowly herbaceous plants. And, secondly, it is no less worthy of remark that the tropical genera include for the most part a greater number of species than those belonging to the extratropical zones; that the polar and circumpolar regions exhibit examples of a greater number of genera in any given number of species, *i. e.* possess fewer species of the genera present in them that either the torrid or the temperate zones, and that it is in the moister parts of the equinoctial, or the temperate regions, and especially in their warmer or subtropical latitudes, that individual plants are the most abundantly produced, and grow to the most excessive size." (P. 1063.)

We have at different times been permitted, by the kindness of Mr. Burnett's publisher, to embellish our pages with several of the cuts which illustrate the Outlines; and we are again under obligations to him for allowing us to show our readers, by the breadth and relief of the following designs, how great has been the progress of xylography within these few years.

*Brugmansia Zippeli.*

(*a*) A fully developed plant, growing parasitically on the root of a cissus, with two others, in a very young state.

(*b c*) Other plants, in further stages of growth, to show the scales by which the flower is enclosed, as by an hybernacle.

*Rafflesia Patma.*

(*a*) Entire flower. (*b*) Section of a bud beginning to expand. (*c*) Another, before expansion.

An extract or two from the Synopsis, with which the work concludes, may give some notion of the classification adopted, and will show that Professor Burnett excels as well in the austerities as in the amenities of botany.

"CLASS VI. PALMARES: *PALMS, and their Allies.*

"(4976.) *SYN.* ARBORES ARUNDINACEÆ, HERBÆ BULBOSÆ et BULBOSIS AFFINES, *Ray.* PALMÆ et LILIA, *Lin.* Part of MONOCOTYLEDONES, *Juss.* Part of ENDORHIZEÆ, *Richard.* ENDOGENÆ PETALOIDEÆ, *De Cand.*, &c. Part of ENDOGENÆ CRYPTOCOTYLEDONEÆ, *Agardh.*

"(4977.) GEN. RULE. Non-glumose flowering Termaffines; or monocotyledonous endogenæ, with the flowers either naked or invested by a distinct perianth, which is often petaloid.

"(4978.) EXCEPTIONS. In some plants, as in Lemna, tubular vessels have not been observed; and in others, as some of the Aroidea, the points of germination are indeterminate.

"(4979.) *Obs.* The stems of the Palmares are in general unbranched, only a single bud being usually developed. They are either abortive, as in the bulbiferous species, or columnar, as in the Palms. Two or more buds are however sometimes developed, as in the garlic, and the stem becomes occasionally branched, as in the rhizoma of the iris, the asparagus, the doum palm, &c. The venation of the leaves is also for the most part simply linear; but in the Smilaceæ, Dioscoraceæ, and Callaceæ, it is retiform: the leaves likewise, which are almost universally without articulation, are distinctly articulated with the stem, in many of the Orchidinæ." (P. 1099.)

"PINEALES.

"(5648.) *SYN.* CONIFERÆ, *Ray, Lin., Juss.*, &c.

"GEN. RULE. Gymnospermous exogenæ with branched stems, simple leaves, linear costules, non-gyrate vernation, and resinous juices. (1400.)

"(5649.) *TAXINÆ.* TAXACEÆ. BACCIFERÆ (part), *Ray.* CONIFERÆ (part), *Juss., De Cand.*, &c.

"GEN. RULE. Ib. The pistilline flowers being distinct, and the fruit a taxule, *i. e.* the solitary seed invested by a succulent scale. (1405.)

"(5650.) *CUPRESSINÆ.* THUJACEÆ. BACCIFERÆ (part), *Ray.* CONIFERÆ (part), *Lin., Juss.*, &c.

"GEN. RULE. Ib. The pistilline flowers being congested and erect, and the fruit a galbule. (1404.)

"(5651.) *ABIENTINÆ.* PINACEÆ.

"GEN. RULE. Ib. The pistilline flowers being congested and reversed, and the fruit a strobile or true scaly cone. (1403.)" (P. 1149.)

## Figures and Tables

**Figure f1:**
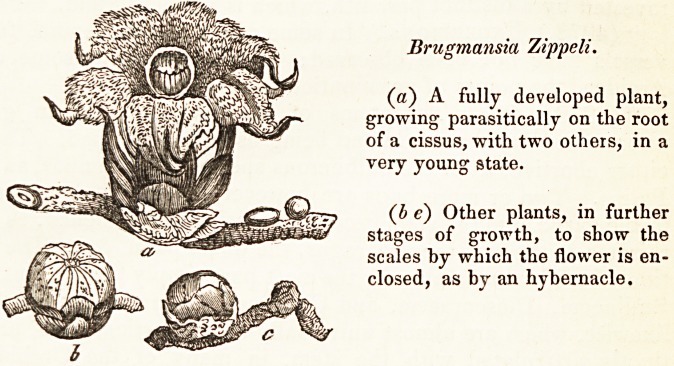


**Figure f2:**